# Insight into the herbicide resistance patterns in *Lolium rigidum* populations in Tunisian and Moroccan wheat regions

**DOI:** 10.3389/fpls.2024.1331725

**Published:** 2024-02-06

**Authors:** Zakia El-Mastouri, Pavlína Košnarová, Kateřina Hamouzová, Ezzedine Alimi, Josef Soukup

**Affiliations:** ^1^ Department of Agroecology and Crop Production, Faculty of Agrobiology, Food and Natural Resources, Czech University of Life Sciences Prague, Prague, Czechia; ^2^ Maghreb Phytotest, Tunis, Tunisia

**Keywords:** *Lolium rigidum* (rigid ryegrass), ALS inhibitor, ACCase inhibitor, herbicide resistance, target site resistance (TSR), non-target-site resistance (NTSR), cytochrome P450

## Abstract

Rigid ryegrass (*Lolium rigidum* Gaud.) is one of the most troublesome weeds in Moroccan and Tunisian cereal crop fields. In total, 19 rigid ryegrass field populations were randomly selected in northern wheat crop areas of Morocco and Tunisia to examine the patterns of herbicide resistance to acetolactate synthase (ALS)- and acetyl-CoA carboxylase (ACCase)-inhibiting herbicides. Greenhouse experiments confirmed reduced sensitivity to ALS- and/or ACCase-inhibiting herbicides in all *L. rigidum* populations. The occurrence of target-site resistance (TSR) was tested using high-throughput genotyping. The advent of next-generation sequencing (NGS) has enabled easy identification of causal mutations and confirmed the presence of ALS and ACCase mutations at specific codons conferring TSR. Thirteen populations showed resistance to ALS-inhibiting herbicides associated with point mutations in positions Pro-197-Thr, Pro-197-Ser, Pro-197-Leu, Pro-197-Gln and Trp-574-Leu, while resistance to ACCase-inhibiting herbicides was detected in 18 populations in positions Asp-2078-Val, Trp-2027-Cys, Ile-1781-Leu, Gly-2096-Ala, and Ile-2041-Asn of the enzymes conferring TSR. Additionally, dose–response experiments with pyroxsulam applied after the inhibition of cytochrome P450 monooxygenase by malathion showed an increase in sensitivity in two out of seven highly resistant (HR) rigid ryegrass populations. This demonstrates the presence of non-target-site resistance (NTSR) in some ryegrass populations. Further evidence of NTSR was investigated in dose–response experiments with pyroxsulam, following pretreatment with the glutathione *S*-transferase (GST) inhibitor 4-chloro-7-nitrobenzoxadiazole (NBD-Cl), which partially reversed resistance in only a few individuals of two *L. rigidum* populations. Hence, our study confirms the existence of multiple and cross-resistance to ALS- and ACCase-inhibiting herbicides in *L. rigidum* from Morocco and Tunisia with both TSR and NTSR mechanisms. These results emphasize local resistance management as an important tool to detect and mitigate gene flow from rigid ryegrass populations where resistance has evolved.

## Introduction

In both of the North African countries Morocco and Tunisia, the cereal sector plays an important role in agricultural production (Lhomme et al., 2009; Latiri et al., 2010). Under rainfed conditions, cereals occupy approximately 1.5 million hectares in Tunisia (Dhehibi et al., 2012; Ben Abdelmalek and Nouiri, 2020; Khammassi et al., 2020) and 5 million hectares in Morocco. Cereal fields are mainly located in the northern part of both countries, but also, to a limited extent, in the arid southern regions.

According to region, small grain cereals are planted annually between October and December, with durum wheat (*Triticum durum*) and soft wheat (*Triticum aestivum*) as the major dominant crops in Tunisia and Morocco, respectively. The farming system is mainly continuous cropping with a very short rotational system. These intensive practices increase management selection pressure, resulting in the emergence of herbicide-resistant populations (Délye, 2005; Košnarová et al., 2021).

Variations in annual cereal production are related to several factors, including the weed species spectrum (Slama et al., 2005; Annabi et al., 2009; Lhomme et al., 2009; Dhehibi et al., 2012; Radhouane, 2013; Khammassi et al., 2020). Rigid ryegrass (*Lolium rigidum* Gaud.) is an annual plant species of the ryegrass genus, which is a highly genetically diverse, cross-pollinated tribe (Heap and Knight, 1986; Bararpour et al., 2017). In Morocco and Tunisia, *L. rigidum* is abundant in all cereal crops but particularly in the irrigated and rainfed areas (480,000 in Tunisia and 2 million hectares in Morocco approximately) (Khammassi et al., 2017; Tanji and Boutfirass, 2018; Khammassi et al., 2020). The introduction of acetolactate synthase (ALS)- and acetyl-CoA carboxylase (ACCase)-inhibiting herbicides was quickly adopted by Tunisian and Moroccan farmers in the 1990s to control ryegrass species and other grasses (Menchari et al., 2016; Bouhache, 2018; Tanji and Boutfirass, 2018; Bouhache, 2020). Herbicides from both sites of action are extensively used by Tunisian and Moroccan growers for several reasons, including: (1) low cost, (2) high efficacy, (3) large spectrum of weeds at very low rates, and (4) environmental safety profile (Khammassi et al., 2020; Tanji et al., 2020). The ACCase herbicides include aryloxyphenoxy-propionates (APPs), cyclohexanediones (DIMs), and the more recent phenylpyrazoline (DEN). The mode of action of this group is selective to several crops and effective on the plastidic homomeric ACCase in grass species, with little to no activity on the heteromeric broad-leaf equivalent ACCase (Délye et al., 2002; Délye, 2005; Khammassi et al., 2020; Jursík et al., 2021). The ALS inhibitor herbicides inhibit the first enzyme in the biosynthetic pathway of branched-chain essential amino acids valine, leucine, and isoleucine. Herbicides in this site of action belong to chemical families of sulfonylureas (SUs), imidazolinones, triazolopyrimidines (types 1 and 2), pyrimidinyl-benzoates, sulfonanilides, and triazolinones. ALS inhibitors are one of the most broadly used classes of herbicides (Green, 2014).

The ryegrass populations in Tunisian and Moroccan cereal fields have varying sensitivity to ALS and ACCase-inhibiting herbicides (Tanji and Boutfirass, 2018; Khammassi et al., 2020). Several herbicide bioassays revealed an increase in resistance levels in both countries (Hajri et al., 2015; Bouhache, 2018; Tanji and Boutfirass, 2018; Bouhache, 2020; Khammassi et al., 2020). The genetic basis of herbicide resistance has not been well documented because of the lack of testing facilities and the high cost.

Herbicide resistance can be classed into two mechanisms: target-site resistance (TSR) and non-target-site resistance. The TSR corresponds to the increased expression of the target protein or structural changes to the herbicide binding. Regarding ALS- and ACCase-inhibiting herbicides, basic biomolecular techniques allowing the detection of known mutation area(s) at one or several loci: 122, 197, 205, 376, 377, 574, 653, and 654 (for ALS) and 1781, 1999, 2027, 2041, 2078, 2088, 2096, and 2097 (for ACCase) are well reported in grass weeds (Délye, 2005; Marshall and Moss, 2008; Powles and Yu, 2010). The use of next-generation sequencing (NGS) has greatly facilitated research (Griffin et al., 2011; Mestanza et al., 2019) related to resistance diagnosis in weed science by allowing massive genotyping-by-sequencing and seeking mutations involved in both ALS- and ACCase-inhibiting herbicides in a large number of ryegrass populations (Chen et al., 2014; Délye et al., 2020; Mohamadi et al., 2022). Non-target-site resistance (NTSR) is the second class of herbicide resistance. This has a broader definition, as it includes multiple mechanisms that could prevent the herbicide from reaching its target (Suzukawa et al., 2021). This also includes the enhanced metabolic resistance (EMR), which is a well-studied mechanism and reported in *L. rigidum* with both ALS- and ACCase-inhibiting herbicides (e.g., Torra et al., 2021). In fact, herbicide metabolism means the degradation of herbicide molecules by specific endogenous plant enzymes (Suzukawa et al., 2021). This type of NTSR is nonspecific, as a single enzyme may inactivate one or more herbicides within the same or different chemical classes (Délye, 2013). Cytochrome P450 monooxygenases (CYP450s) are one of the most well-known enzyme classes identified in the process of herbicide detoxification. In fact, the increase in the activity of this enzyme can lead to metabolism-based herbicide resistance (Siminszky, 2006).

In addition to P450s, glutathione *S*-transferase (GST) is an important enzyme that metabolizes herbicide molecules. It seems to be involved in cutting herbicide bounds by conjugating glutathione with herbicide molecules and facilitating the recognition of glutathione transporters, which leads to reduced translocation (Suzukawa et al., 2021). Therefore, the GST biochemical activity assay and compounds such as GST inhibitor 4-chloro-7-nitrobenzoxadiazole (NBD-Cl) are important tools to highlight the involvement of GSTs in weed herbicide resistance (Cai et al., 2022).

In this paper, we aim to (1) identify herbicide resistance to ALS and ACCase using preselection phenotypic data and (2) investigate the molecular basis of resistance to ALS- and ACCase-inhibiting herbicides in *L. rigidum* populations collected from Morocco and Tunisia.

## Materials and methods

### Plant material

**Figure 1:**
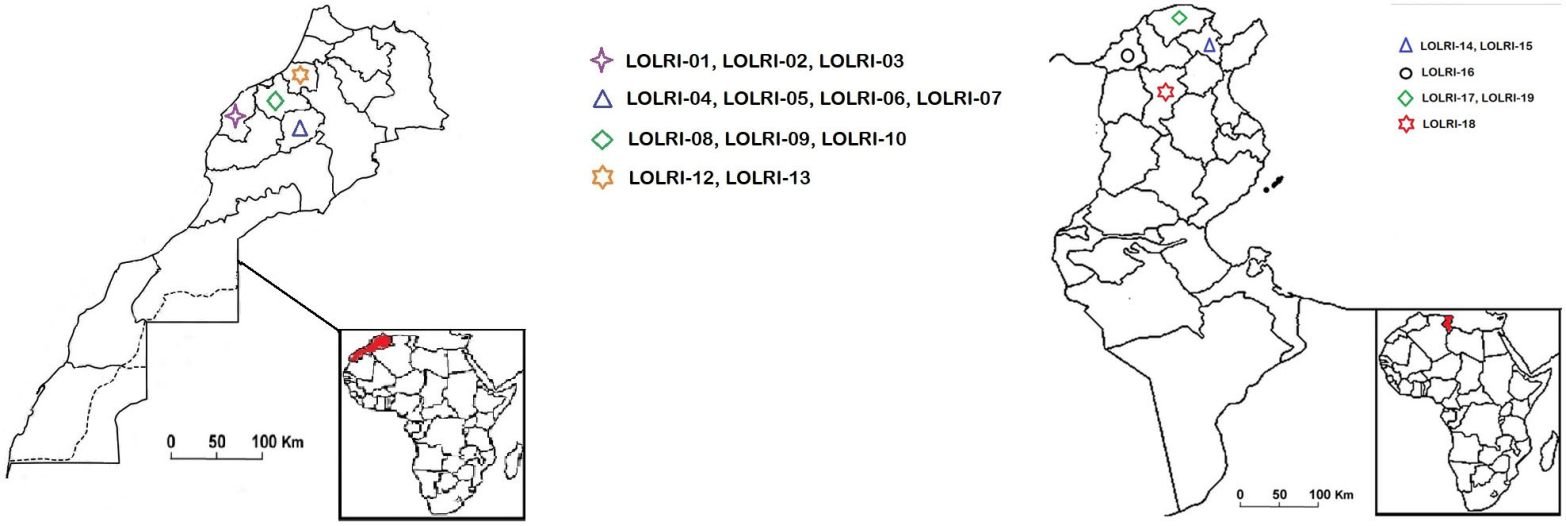
Maps of Tunisia (left) and Morocco (right) showing regions where susceptible ryegrass populations were collected. Populations one to 13 are related to Morocco, and populations 14 to 19 are referring to Tunisian areas.

A total of 20 ryegrass populations (hereafter named LOLRI-01–LOLRI-19, LOLRI-S) collected from both northern Tunisia and Morocco cereal field regions were tested for resistance to ALS- and ACCase-inhibiting herbicides (**Figure 1**; **Table 1**). The fields sampled had a history of ALS- and ACCase-inhibiting herbicide applications against ryegrass, but the details of this history could not be obtained. Wheat was usually the main crop, with no intercropping. Diclofop, fenoxaprop, fluazifop, imazamethabenz, tralkoxydim, etc. were largely the oldest ALS- and ACCase-inhibiting herbicides applied in these fields. Seeds of *L. rigidum* were collected homogenously from at least 250 plants in each field at weed maturity (May to July 2021). An additional common population of ryegrass (hereafter named LOLRI-S) was taken from the Atlas Mountains that stretch over 2,500 km (about half the width of the USA) from Morocco to Tunisia as a susceptible species with no herbicide history (35.0391N, 0.2909E). The germination rate of all populations was 95%, including the sensitive population, at the time of pot experiment establishment.

**Table 1:** Herbicides and rates used in the dose–response study with *Lolium rigidum*.

Local trade name	Groupe HRAC	Site of action	Active ingredient	g a.i L^−1^ or g a.i kg^−1^	Local registered rate (N)	Gradient tested^a^
**Pallas OD**	2	Inhibition of ALS	Pyroxsulam + safener	45 g a.i L^−1 ^+ 90	0.5 L ha^−a^	NT, N/2, N, 2N
**Axial EC**	1	Inhibition of ACCase	Pinoxaden + safener	45 g a.i L^−1 ^+ 11.25	1 L ha^−a^	NT, N/2, N, 2N
**Brocar EC**	1	Inhibition of ACCase	Clodinafop + safener	240 g a.i L^−1 ^+ 60	0.25 L ha^−a^	NT, N/2, N, 2N
**Amilcar/Cossack OD**	2	Inhibition of ALS	Iodosulfuron + mesosulfuron + safener	7.5 g a.i L^−1 ^+ 7.5 g a.i L^−1 ^+ 22.5	1.25 L ha^−a^	NT, N/2, N, 2N

NT, nontreated; N, recommended field dose registered in both Morocco and Tunisia; HRAC, Herbicide Resistance Action Committee.

a Applied to 19 resistant and one sensitive populations as first prescreening.

### Dose–response trial

Seeds from all populations listed above were sown directly in plastic pots (diameter: 70 mm, height: 90 mm) filled with potting mix (pH = 7.5, 1% organic matter, clay containing chernozem, 46% loam, 209 mmol (+) sorption capacity, 87 ppm P, 203 ppm K, 197 ppm Mg, 8.073 ppm Ca), with 20 seeds per pot, and covered with 0.5 cm high potting mix. Pots were randomly placed in the greenhouse located at the Czech University of Life Sciences Prague (49.0915833N, 14.7343022E) under a determined temperature (22°C/18°C) and light (14 h/10 h), respectively. Plants were irrigated using tap water regularly, with fertilization (N-P-K, 7-7-6) according to the need. At the one- to two-leaf growth stage, plants were thinned to leave 10 plants per pot for herbicide treatment and evaluations.

Four formulated herbicides were included in the trial, as was an untreated control. All herbicides were applied postemergence (BBCH 12–13) according to the label (**Table 1**). Treatments were arranged in a randomized complete block design with four replications. Pots were arranged in specific plates as an experimental unit, depending on dose and herbicide application. Plates were subsequently randomized with respect to replicates and populations. The applications were made using a laboratory spray chamber set up with Lurmark 01F80 nozzles to deliver 250 L/ha at a pressure of 250 Pascal (30 psi).

The mean temperatures during the 28 days following spraying ranged ± 22.3°C by day and ±14.2°C at night. The air humidity ranged between 45% and 65% (mean relative humidity for 24 h). Efficacy was evaluated as a visual percentage control based on a 0%–100% linear scale, assessed at 28 days after treatment (DAT). The number of surviving plants was recorded, as were the shoot fresh and dry weights. The experiment was repeated twice in space and time.

### ALS and ACCase molecular assays: next-generation sequencing

Leaf samples of *L. rigidum* populations were collected from the ALS and ACCase -inhibitor sensitivity screening. Within each population, one disc of a 2-mm diameter hollow punch was used. A total of 50 leaves (individuals) representing each ryegrass population were punched and placed in a 2-mL microtube containing two steel beads. Tubes were kept open for at least 2 weeks at room temperature to let samples dry and prevent molding before sending them to INRAe (Dijon, France) for TSR genotyping using Illumina NGS technology. Several regions of the ACCase and ALS genes were amplified separately from each DNA extract primer pairs as detailed by Délye et al. (2020). These regions carried the eight codons involved in TSR to ALS inhibitors (codons 122, 197, 205, 376, 377, 574, 653 and 654, as standardized according to the *Arabidopsis* ALS sequence) and seven additional codons potentially involved in TSR (codons 121, 124, 196, 199, 375, 571 and 578) were included in this genetic diagnosis (Délye et al., 2020). The codons with a demonstrated role in resistance to one or more ACCase -inhibiting herbicides in field weeds are: 1781, 1999, 2027, 2041, 2078, 2088, 2096 (Délye et al., 2002). As for ALS, about fifteen additional codons located in regions of ACCase interacting with herbicides were also analyzed and to which no role in weed resistance has been demonstrated. Sequencing and data analysis were performed as described by Délye et al.,2020. The raw sequence data have been deposited with links to BioProject accession number PRJNA1045233 in the NCBI BioProject database (http://www.ncbi.nlm.nih.gov/bioproject/ www.ncbi.nlm.nih.gov/bioproject/).

### Response of whole plants to herbicide in combination with malathion

Seeds from *L. rigidum*, including the sensitive population, were sown, grown, and kept in the same conditions as described above. To highlight the role of P450, its inhibition is induced before herbicide application (Busi et al., 2011). The first treatment was an application of pyroxsulam (Pallas OD), at BBCH 12–13. In the second treatment, pyroxsulam was applied 1 h after the application of malathion at 0 or 1,000 g ai ha^−1^ (organophosphate insecticide, CYP450 inhibitor). Pyroxsulam doses applied postemergence were 0, 1.4625 g ai ha^−1^, 5.625 g ai ha^−1^, 11.25 g ai ha^−1^, 22.5 g ai ha^−1^ (field rate), and 45 g ai ha^−1^. The experimental layout was as described in the preceding dose–response screening. The fresh weight of aboveground tissue was collected at 28 DAT, and survivors were counted in each pot.

### Response of whole plants to herbicide in combination with NBD-Cl

Plant materials from *L. rigidum* populations were prepared as described in dose–response trial. A known GST inhibitor, NBD-Cl, has been used as a tool to highlight the role of GST in herbicide resistance (Cai et al., 2022). One hour before pyroxsulam application, 270 g ai ha^−1^ of NBD-Cl was applied at the BBCH 12–13 growth stage. Three pyroxsulam rates of 11.25 g ai ha^−1^, 22.5 g ai ha^−1^, and 45 g ai ha^−1^ were applied. The experimental layout was as described in the preceding dose–response screening. Survival data were collected by counting the number of surviving individuals in each pot at 28 DAT.

### Statistical analysis

For the prescreening experiments, the survival percentage for each *L. rigidum* population and each herbicide (ALS and ACCase inhibitors) was analyzed using a linear model (two-way ANOVA) with R (v.3.5.3) software. Populations and herbicide rates were considered factors and replicates an error term (block). The reliability of the ANOVA model was checked by four different plots: (1) Q–Q or quantile–quantile plot, (2) residual versus fitted plot, (3) scale-location plot, and (4) Cooks distance plot. In addition, a Shapiro–Wilk test was assessed for normality, and data were transformed when necessary. Tukey’s honest significant difference (HSD) tests were performed to compare the percentage control of suspected resistant populations to the known susceptible population at a probability level of 0.05.

Dose–response analyses with the application of malathion in POST were performed by analyzing survival percentage and fresh biomass for each population and each herbicide. The four-parameter Log-logistic models were assessed, and the normality of residuals was checked. GR_50_ and GR_90_ were calculated and corresponded to rates that required 50% and 90% survivor reduction, respectively. For some populations, GR_50_ could not be calculated because the survival percentage did not decrease by 50% even at the highest rates (× 50 field rate tested separately). The resistance index (RI) was calculated by dividing GR_50_ or GR_90_ values for each population tested by the same parameters extracted from the sensitive population (LOLRI-S), respectively. The dose–response studies were repeated twice, and data were pooled before analysis.

## Results

### Prescreening and dose–response results

**Figure 2:**
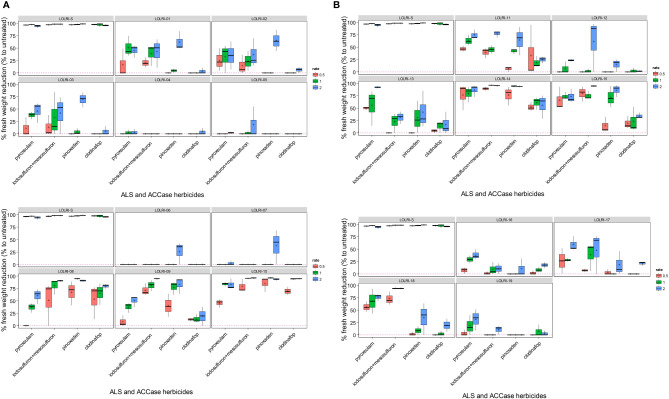
**(A)** Boxplots of reduction in foliage fresh biomass relative to untreated control for suspected ALS- and ACCase-inhibiting resistant *Lolium rigidum* from 1 to population 10, including sensitive (LOLRI-S). Four herbicides were applied at three rates each from left to right (orange, green, and blue, respectively) with pyroxsulam (11.25 g ha^−1^, 22.5 g ha^−1^, and 45 g ha^−1^), iodosulfuron + mesoulfuron (4.7 + 4.7 g ha^−1^, 9.4 + 9.4 g ha^−1^, and 18.8 + 18.8 g ha^−1^), pinoxaden (25 g ai ha^−1^, 50 g ha^−1^, and 150 g ai ha^−1^), clodinafop (30 g ai ha^−1^, 60 g ai ha^−1^, and 120 g ai ha^−1^). The median is represented by the horizontal line inside boxes, and the short lines outside boxes correspond to minima and maxima. **(B)** Boxplots of reduction in foliage fresh biomass relative to untreated control for suspected ALS- and ACCase-inhibiting resistant *Lolium rigidum* from 11 to population 19, including sensitive (LOLRI-S). Four herbicides were applied at three rates each from left to right (orange, green, and blue, respectively) with pyroxsulam (11.25 g ai ha^−1^, 22.5 g ai ha^−1^, and 45 g ai ha^−1^), iodosulfuron + mesoulfuron (4.7 + 4.7 g ai ha^−1^, 9.4 + 9.4 g ai ha^−1^, and 18.8 + 18.8 g ai ha^−1^), pinoxaden (25 g ai ha^−1^, 50 g ai ha^−1^, and 150 g ai ha^−1^), clodinafop (30 g ai ha^−1^, 60 g ai ha^−1^, and 120 g ai ha^−1^). The median is represented by the horizontal line inside boxes, and the short lines outside boxes correspond to minima and maxima.

The tested ACCase- and ALS-inhibiting herbicides showed low control in almost all selected, suspected resistant, populations of *L. rigidum* (**Figures 2A, B**) at all rates in a comparison with a sensitive population (LOLRI-S). Fresh biomass was used as a response parameter to reflect the real physiological activity of leaves associated with their photosynthesis and respiration better than dry biomass (Huang et al., 2019). Within each population, a significantly lower percentage of fresh biomass was correlated to the rate of each herbicide tested (*R*² = 0.62, *p*-value <0.001). Fresh biomass reduction varied significantly between all ACCase- and ALS-inhibiting herbicides tested (*F*-value = 9.76, *p*-value <0.001) and ryegrass populations (*F*-value = 61.73, *p*-value <0.001). However, there was no significant interaction (at *α* = 0.05) between herbicide treatment (rate) and population (*F*-value = 1.41, *p*-value = 0.052), which confirmed that both parameters are independent. In other words, the variation in the percent reduction of fresh biomass was due to differences in plant populations rather than herbicide doses.

All ryegrass populations showed low control by one or both ACCase- and/or ALS-inhibiting herbicides tested at a double field rate (<10% of fresh biomass reduction) except LOLRI-08, LOLRI-09, LOLRI-10, LOLRI-13, LOLRI-14, LOLRI-15, and LOLRI-18, which showed high sensitivity to at least one of the four herbicides tested (**Figures 2A, B**). Populations LOLRI-10 and LOLRI-14 were highly sensitive to three of the four herbicides tested. Their responses were similar to the untreated check, LOLRI-S (*p*-value = 0.97, *p*-value = 0.93, respectively), followed by LOLRI-15 (*p*-value = 0.15). However, populations LOLRI-04, LOLRI-05, LOLRI-06, LOLRI-07, and LOLRI-19 were highly resistant (HR) to all herbicides tested (<25% fresh biomass reduction at double field rate). Fresh weight reductions at field rate (N) were 25% and 40% with ACCase- and ALS-inhibiting herbicides, respectively. Except for LOLRI-10, all Moroccan ryegrass populations were HR to clodinafop (0% fresh weight reduction at double field rate 120 g ai ha^−1^; also check this error throughout the paper). However, for the same active ingredient, the Tunisian populations showed a higher degree of fresh weight reductions (20%–40%) at a double field rate than the Moroccan populations (**Figure 2B**). Some ryegrass populations showed more sensitivity to pyroxsulam at both field and double field rates. Pinoxaden provided low control of the Tunisian populations (except LOLRI-15 and LOLRI-16) versus Moroccan ones. With both pinoxaden and iodosulfuron + mesosulfuron, individuals from each ryegrass population reacted differently (**Figures 2A, B**). These results suggested a reduction in sensitivity to ACCase- and/or ALS-inhibiting herbicides in individuals within all ryegrass populations tested.

### Target-site resistance: next-generation sequencing

For a better understanding of mechanisms endowing resistance to both ALS- and ACCase-inhibiting herbicides, the detection of mutations in codons of interest was investigated by NGS technology (ALS and ACCase molecular assays: next-generation sequencing). From all *L. rigidum* populations tested, 14 populations possessed ALS mutations (73.6%), 18 possessed ACCase mutations (94.7%), and 13 showed mutations on the two genes (68.4%, **Table 2**).

**Table 2:** Mutations in 13 Moroccan (LOLRI-01 to LOLRI-13) and six Tunisian (LOLRI-14 to LOLRI-19) ryegrass populations analyzed.

ALS mutations	ACCase mutations	New mutations	Populations where found
Trp-574-Leu, Pro-197-Thr	Asp-2078-Val, Ile-1781-Leu, Ile-2041-Asn	ND	LOLRI-01
Pro-197-Gln, Trp-574-Leu	Ile-1781-Leu, Asp-2078-Val, Trp-2027-Cys, Ile-2041-Asn	ND	LOLRI-02
Pro-197-Thr	Asp-2078-Val, Ile-1781-Leu	ND	LOLRI-03, LOLRI-18
Pro-197-Ser, Pro-197-Leu	Ile-1781-Leu, Asp-2078-Val, Ile-2041-Asn	ND	LOLRI-04, LOLRI-17
Trp-574-Leu, Pro-197-Ser, Pro-197-Leu, Pro-197-Thr, Pro-197-Gln	Ile-1781-Leu, Ile-2041-Asn	ND	LOLRI-05
Pro-197-Ser, Pro-197-Gln, Pro-197-Thr, Trp-574-Leu	Ile-1781-Leu, Gly2096-Ala, Ile-2041-Asn	Gly-1706-Asp	LOLRI-06
ND	Ile-1781-Leu, Ile-2041-Asn	ND	LOLRI-07, LOLRI-19
Pro-197-Gln	ND	ND	LOLRI-08
ND	Ile-1781-Leu, Trp-2027-Cys	ND	LOLRI-09
Trp-574-Leu	Asp-2078-Val	ND	LOLRI-10
ND	Asp-2078-Val, Ile-2041-Asn, Ile-1781-Leu	ND	LOLRI-11, LOLRI-13, LOLRI-14
Pro-197-Thr, Pro-197-Gln	Ile-1781-Leu, Trp-2027-Cys, Ile-2041-Asn, Asp-2078-Val	ND	LOLRI-12, LOLRI-16
Pro-197-Thr	Asp-2078-Val, Ile-2041-Asn, Ile-1781-Leu	ND	LOLRI-15

ND, not detected.

Regarding ALS-inhibiting herbicides, 13 populations possessed one to four mutations at codon 197 (Ser-197, Leu-197, Thr-197, and Gln-197); four of the six possible single mutations at codon 197 were identified. In addition, mutations at codon 574 were also detected in six *L. rigidum* populations (Leu-574), five of which also had one or more mutations at codon 197 (**Table 2**). Regarding ACCase-inhibiting herbicides, 17 populations possessed mutations detected at codon 1781. The mutation detected at codon 2027 was found in four *L. rigidum* populations. Codon 2041 mutations were detected in 14 populations. At codon 2078, 11 populations of 18 tested possessed mutations. Only one population (LOLRI-06) showed mutation at codon 2096. A new mutation was detected at codon 1706 (Gly to Asp) in LOLRI-06, where three other mutations known to give resistance to ACCase inhibitors were also present (Ile-1781-Leu, Gly-2096-Ala, and Ile-2041-Asn). The identification of the presence of both ALS- and ACCase-inhibiting herbicide-available mutations corresponded with the survivor of all herbicide-treated plants tested for target site mutation surviving herbicide treatment in these populations. The sensitive population LOLRI-S had no mutations, and all herbicide-treated plants were recorded as dead at 28 DAT.

### Non-target-site resistance: CYP450 and GST assays

#### CYP450: determination of herbicide efficacy in a synergism with malathion

**Figure 3:**
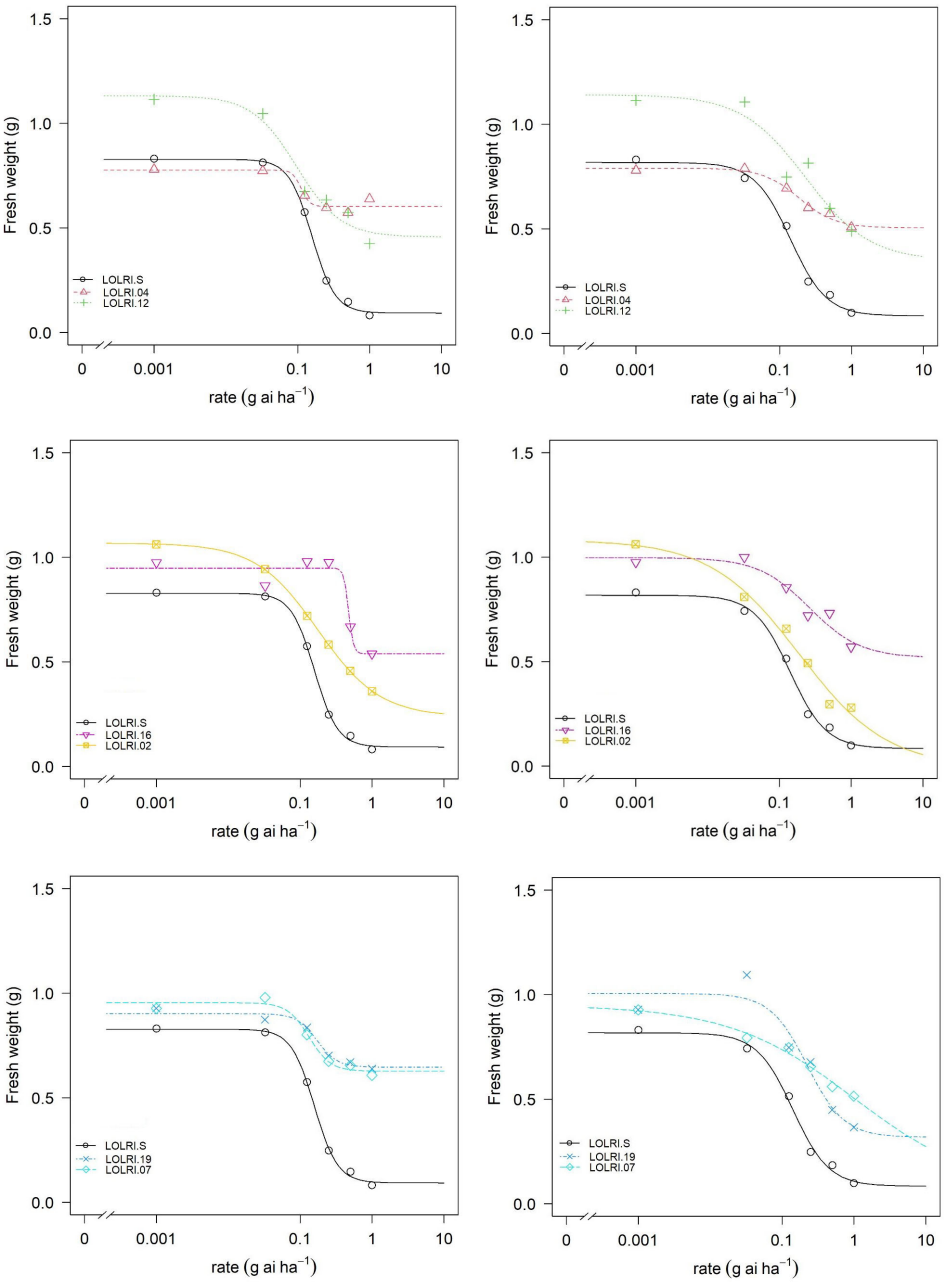
Dose–response curves (log-logistic four-parameter function) for two Tunisian (LOLRI-16 and LOLRI-19) and four Moroccan (LOLRI-02, LOLRI-04, LOLRI-07, and LOLRI-12) *Lolium rigidum* populations treated with pyroxsulam without malathion (left) and in mixture with malathion at 1,000 g ai ha^−1^ (right). Dark lines and circles represent the sensitive population (LOLRI-S).

In the seven populations randomly selected (LOLRI-02, LOLRI-04, LOLRI-07, LOLRI-12, LOLRI-16, LOLRI-19, and LOLRI-S), the application of malathion alone did not impact all measured parameters (fresh weight, dry weight, and percentage of survivor). Also, the application of pyroxsulam with malathion did not show any difference if compared with pyroxsulam, applied alone, in all measured parameters with sensitive population LOLRI-S (**Table 3**; **Figure 3**). In populations LOLRI-04 and LOLRI-12, both GR_50_ and GR_90_ showed slight differences when malathion was sprayed before the herbicide application, which led to the conclusion that CYP450 has no role in enhanced metabolism in these populations (**Table 3**; **Figure 3**). In LOLRI-02, LOLRI-16, and LOLRI-19, both RI_50_ and RI_90_ of percent survivors decreased (12.11 and 2.1, respectively), with a large difference observed in LOLRI-19 (8.26 and 4.42, respectively). LOLRI-07 showed a decreased slope as well as a significant effect of the P450 inhibitor while mixed with pyroxsulam (*p*-value = 0.016).

**Table 3:** Values of the log-logistic models from dose–responses using pyroxsulam (± malathion) at 1,000 g ai ha^−1^ of two Tunisian (LOLRI-16, LOLRI-19) and four Moroccan (LOLRI-02, LOLRI-04, LOLRI-07, and LOLRI-12) *Lolium rigidum* populations and one sensitive reference population (LOLRI-S).

Parameter	Population	Treatment	Slope ^a^	GR_50_	GR_90_	RI_50_^b^	RI_90_^c^	Std. error^c^	Std. error^d^	*p*-value^e^
Survival (%)	LOLRI-S	− Malathion	0.091	0.10	0.05	1.00	1.00	0.00	0.01	1.12*E*−13
	LOLRI-S	+ Malathion	0.081	0.08	0.05	1.00	1.11	0.01	0.01	7.61*E*−08
	LOLRI-04	− Malathion	0.224	–^f^	0.21	–^f^	4.19	–^f^	0.12	0.13
	LOLRI-04	+ Malathion	0.251	–^f^	0.31	–^f^	6.21	–^f^	0.05	2.26*E*−08
	LOLRI-12	− Malathion	0.945	3.26	0.20	34.21	4.14	4.81	0.13	0.37
	LOLRI-12	+ Malathion	10.143	4.59	0.27	56.14	5.43	9.59	0.48	0.65
	LOLRI-16	− Malathion	0.500	–^f^	0.43	–^f^	8.66	–^f^	0.06	3.96*E*−10
	LOLRI-16	+ Malathion	0.172	–^f^	0.14	–^f^	2.86	–^f^	0.02	3.83*E*−05
	LOLRI-02	− Malathion	0.278	0.59	0.14	6.20	2.85	0.07	0.01	7.82*E*−14
	LOLRI-02	+ Malathion	4.706	0.43	0.03	5.29	0.63	0.86	0.04	0.71
	LOLRI-19	− Malathion	8.218	1.94	0.32	20.37	6.52	8.49	1.26	0.84
	LOLRI-19	+ Malathion	0.538	0.68	0.22	8.26	4.42	0.11	0.02	2.59*E*−07
	LOLRI-07	− Malathion	12.202	1.93	0.10	20.28	2.06	3.69	0.17	0.65
	LOLRI-07	+ Malathion	0.303	–^f^	0.17	–^f^	3.53	–^f^	0.04	0.016

GR_50_ and GR_90_: herbicide concentration that required for 50% and 90% of *L. rigidum* survivor reduction, respectively. RI: Resistance Index calculated as ^(b)^ RI_50_=GR_50_(R)/GR_50_(S) or ^(c)^ RI90=GR_90_(R)/GR_90_(S). ^(a)^ Slope at GR_50_. ^(d) (e)^ Standard errors at GR_50_ and GR_90_, respectively. ^(f)^ P-value at GR_50_. ^(g)^ values can not be estimated.

**Figure 4:**
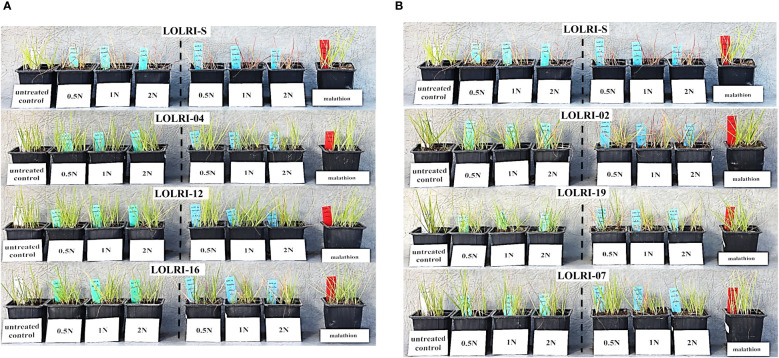
**(A)** Phenotype results of two Moroccan populations (LOLRI-04 and LOLRI-12), one Tunisian population (LOLRI-16), and sensitive *Lolium rigidum* (LOLRI-S) 28 days after application of pyroxsulam at 11.25 g ai ha^−1^ (0.5N), 22.5 g ai ha^−1^ (1N), and 45 g ai ha^−1^ (2N) without malathion in the left (from the second pot with green stickers) and the same rates of pyroxsulam with malathion at 1,000 g ai ha^−1^ in the right (from the fifth pot with blue stickers). First and eighth pots (from left to right) correspond to untreated (white sticker) and malathion (red sticker) modalities at 1,000 g ai ha^−1^, respectively. **(B)** Phenotype results of two Moroccan populations (LOLRI-02 and LOLRI-07), one Tunisian population (LOLRI-19), and sensitive *Lolium rigidum* (LOLRI-S) 28 days after application of pyroxsulam at 11.25 g ai ha^−1^ (0.5N), 22.5 g ai ha^−1^ (1N), and 45 g ai ha^−1^ (2N) without malathion in the left (from the second pot with green stickers) and the same rates of pyroxsulam with malathion at 1,000 g ai ha^−1^ in the right (from the fifth pot with blue stickers). First and eighth pots (from left to right) correspond to untreated (white sticker) and malathion (red sticker) modalities at 1,000 g ai ha^−1^, respectively.

Regarding phenotypic results, most individuals with LOLRI-S had died at all pyroxsulam rates by 28 days after application (with and without malathion) or had severe red leaf symptoms (**Figures 4A, B**). Both LOLRI-04 and LOLRI-12 do not show any impact of the herbicide alone or in combination with the P450 inhibitor (**Figure 4A**). However, individuals of LOLRI-16, LOLRI-02, LOLRI-19, and LOLRI-07 are more sensitive (red leaf symptoms) when malathion is applied at 1,000 g ai ha^−1^ in a mixture with pyroxsulam compared to treatments involving pyroxsulam alone at the same rates (**Figures 4A, B**). Visually, LOLRI-02 seemed to have a high proportion of sensitive individuals (red leaves leading to dead plants) in all rates of pyroxsulam when mixed with malathion (**Figure 4B**) compared to the application of pyroxsulam alone at the same rates. LOLRI-07 showed a slight effect of the P450 inhibitor among the individuals. This suggests that LOLRI-02, LOLRI-16, LOLRI-19, and LOLRI-07 may have the beginnings of a NTSR phenomenon with the P450 mechanism.

#### Glutathione *S*-transferase: determination of herbicide efficacy with inhibited GST

Based on phenotyping results from the pyroxsulam dose–response bioassay, including the GST inhibitor, none of the six resistant *L. rigidum* populations selected (LOLRI-02, LOLRI-04, LOLRI-07, LOLRI-12, LOLRI-16, and LOLRI-19) showed a decrease in fresh weight. The addition of NBD-Cl inhibitor at 270 g ai ha^−1^, when added to all pyroxsulam rates (11.25 g ai ha^−1^, 22.5 g ai ha^−1^, and 45 g ai ha^−1^), did not show much visual impact in all populations except in the sensitive LOLRI-S, as well as very few individuals of LOLRI-02, LOLRI-07, LOLRI-16, and LOLRI-19 (**Table 4**). This leads us to conclude that with pyroxsulam, there might be involvement of glutathione *S*-transferase in the NTSR pathway, but only in some individuals of these four *L. rigidum* populations.

**Table 4:** Percentage of survivals in seven *Lolium rigidum* populations after application of pyroxsulam ± NBD-Cl at 270 g ai ha^−1^.

Pyroxsulam (g ai ha^−1^)	NBD-Cl	LOLRI-S	LOLRI-02	LOLRI-04	LOLRI-07	LOLRI-12	LOLRI-16	LOLRI-19
0	−	100 ± 0	100 ± 0	100 ± 0	100 ± 0	100 ± 0	100 ± 0	100 ± 0
	+	100 ± 0	100 ± 0	100 ± 0	100 ± 0	100 ± 0	100 ± 0	100 ± 0
11.25	−	30 ± 10	100 ± 20	100 ± 0	100 ± 0	100 ± 0	100 ± 0	100 ± 0
	+	30 ± 10	100 ± 20	100 ± 0	100 ± 0	100 ± 0	100 ± 0	100 ± 0
22.5	−	0 ± 0	90 ± 10	100 ± 0	100 ± 10	100 ± 0	90 ± 20	100 ± 10
	+	0 ± 0	90 ± 10	100 ± 0	100 ± 10	100 ± 0	90 ± 20	100 ± 10
45	−	0 ± 0	90 ± 20	100 ± 0	100 ± 10	100 ± 0	90 ± 20	90 ± 0
	+	0 ± 0	90 ± 20	100 ± 0	100 ± 10	100 ± 0	90 ± 20	90 ± 0

## Discussion

Based on phenotype and genotype results and in order to investigate different resistance mechanisms, the populations of *L. rigidum* collected in different locations across Morocco and Tunisia showed resistance to pyroxsulam, mesosulfuron + iodosulfuron, pinoxaden, and clodinafop. Resistance of ryegrass to the same MoA and or SoA was already documented in both Tunisia and Morocco (Bouhache, 2020; Khammassi et al., 2020; Tanji et al., 2020). Other studies confirmed the increase of resistant *L. rigidum* populations to ALS- and ACCase-inhibiting herbicides in wheat fields in the Northern part of Tunisia (Menchari et al., 2016).

Resistance to chemical classes of ALS-inhibiting herbicides in *L. rigidum* can be potentially based on TSR and/or NTSR mechanisms (Busi et al., 2018). By using NGS sequencing, all known ALS and ACCase mutation points were studied and tested in all populations, and the TSR-conferring mutation/s was/were identified (**Table 2**). Six out of 19 populations did not show any ALS mutations, and only one population showed no ACCase TSR-conferring mutations (**Table 2**). From these seven populations, two (LOLRI-02 and LOLRI-19) were revealed to be more sensitive when malathion or NBD-Cl were used with pyroxsulam (**Tables 3**, **4**; **Figures 2.1**, **2.2**). Regarding ALS-inhibiting herbicides, the frequency of mutations in populations ranged from 2% to 91%, with an average of 24.3% (**Table 5**). At codon 197, mutations confer high resistance to sulfonylureas, and a variable level of resistance to other families of ALS inhibitors (Loubet et al., 2021). In addition, codon 574 confers high resistance to all ALS inhibitors (Heap, 2021). Regarding ACCase-inhibiting herbicides, the frequency of mutations in populations was 3% to 162%, with an average of 64.8%. The frequency of ACCase mutations exceeded 100% in several populations with 1781 and 2078 or 1781 and 2041 (**Table 5**). This can be explained by two hypotheses: (1) the presence of double mutations (impossible to be checked with Illumina reads because these two mutations are not carried by the same amplicon), and (2) the presence of *Lolium* polyploid in the populations concerned, although this could not be investigated in the present work. The most frequently detected mutation in all ryegrass populations was at Leucine-1781 (**Table 5**). This codon did confer resistance to most ACCase inhibitors and low resistance to clethodim (Kaundun et al., 2013). The mutation detected at Cysteine-2027 confers resistance to APPs and pinoxaden and was found in four *L. rigidum* populations. Ile-to-Asn substitution at codon position 2041 mutations were detected in 14 populations, and according to bibliography (e.g., Délye et al., 2011; Scarabel et al., 2011; Guo et al., 2017), this codon confers resistance to APPs with weaker resistance to pinoxaden. At codon 2078, the mutation detected confers resistance to all ACCase inhibitors (Délye et al., 2008). Only one population showed mutation at codon 2096. This codon conferred resistance to fops, weak resistance to clethodim, and no resistance to cycloxydim or pinoxaden. An unprecedented mutation was detected at codon 1706 (Gly to Asp) in low frequency (3%, **Table 5**); this mutation is in the herbicide-binding zone of ACCase. Its possible role in the resistance remains to be demonstrated. These frequencies are correlated with the phenotypic results (*R*² = 0.41). Although LOLRI-10 showed mutations at both 2078 and 574 codons (**Table 2**), the frequency of mutation revealed very low (2% and 3%, respectively, **Table 5**), which explains its high phenotypic sensitivity to both ALS and ACCase inhibitors used (**Figures 2.1**, **2.2**).

**Table 5:** Frequency of mutations (%) in 19 ryegrass populations (LOLRI-01 to LOLRI-13 Moroccan and LOLRI-14 to LOLRI-19 Tunisian) by next-generation sequencing.

Population number	ALS					ACCase					Gly-1706-Asp
	Pro-197-Thr	Pro-197-Ser	Pro-197-Leu	Pro-197-Gln	Trp-574-Leu	Ile-1781-Leu	Trp-2027-Cys	Ile-2041-Asn	Asp-2078-Val	Gly-2096-Ala	
LOLRI-01	5				70	45		13	55		
LOLRI-02				11	11	99	25	3	35		
LOLRI-03	22					61			50		
LOLRI-04		41	30			88		3	3		
LOLRI-05	1	13	3	1	62	59		7			
LOLRI-06	7	27		10	3	40		7		6	3
LOLRI-07						99		11			
LOLRI-08				9							
LOLRI-09						31	11				
LOLRI-10					2				3		
LOLRI-11						4		10	18		
LOLRI-12	23			3		15	13	9	6		
LOLRI-13						4		18	18		
LOLRI-14						2		16	15		
LOLRI-15	9					3			4		
LOLRI-16	13			7		21	14	12	3		
LOLRI-17		15	76			41		15	21		
LOLRI-18	12					83			45		
LOLRI-19						111		21			

NTSR is considered to be the predominant type of resistance to both the first and second groups of herbicides worldwide in grasses (Suzukawa et al., 2021) and develops independently from TSR. Our study is the first confirmed report of NTSR in *L. rigidum* in Morocco and Tunisia. In dose–response experiments, pretreatment with the P450 inhibitor malathion had no effect on the efficacy of pyroxsulam in two out of six randomly selected populations. However, it partially synergized in four *L. rigidum* populations, leading to a shift toward sensitivity in the three resistant populations. This indicates that P450 could be involved in the resistance of *L. rigidum* to ALS-inhibiting herbicides, at least those of the sulfonamide family. The CYP450 superfamily is the largest enzymatic protein family in plants, linked to vital functions and participating in the synthesis of fatty acids, sterols, and hormones. Members of this superfamily are involved in multiple metabolic pathways with distinct and complex functions, mediating a vast array of reactions (Siminszky, 2006). Malathion has an inhibiting effect on P450 in plants, which can no longer catalyze herbicide degradation. Therefore, resistant plants may totally or partially, as in the case of this study, lose their resistance.

In addition to cytochrome P450, GSTs are another key enzyme superfamily with many roles in primary and secondary metabolism. Some GST members are involved in herbicide detoxification (Cummins et al., 2013; Cai et al., 2022). Both constitutive and induced GST overexpressions were identified in numerous herbicide-resistant weed species. According to our study, the GST inhibitor NBD-Cl could partially sensitize selected *L. rigidum* populations to pyroxsulam, suggesting GSTs may contribute to these populations’ pyroxsulam resistance. This result was demonstrated in only a few individuals from some populations in the dose–response experiments (**Table 4**). From this, quantifying the GST activity (with the same principle as P450) was difficult to assess in the case of the populations selected.

Cross- and multiple resistances in weeds severely limit herbicide treatment options (Torra et al., 2021). While TSR is logically limited to herbicides of the same mode of action, NTSR mechanisms with dissipated and/or “general use” metabolic proteins are capable of detoxifying a range of herbicides (Siminszky, 2006; Suzukawa et al., 2021).

## Conclusion

To better understand the nature of resistance in Morocco and Tunisia *L. rigidum* populations, we subjected them to the most frequently used active ingredients (pyroxsulam, iodosulfuron + mesosulfuron, pinoxaden, and clodinafop), to which farmers are claiming weed control failure in wheat fields. The 19 populations tested from Morocco and Tunisia wheat fields showed the presence of both TSR (in all populations) and involvement of NTSR (in a few studied populations) mechanisms. Some populations can still be controlled by using ALS and not ACCase herbicides, or vice versa. This leads to confirming that growers can still use both modes of action according to the nature of the resistance involved in their populations. However, the herbicide mode of action used should be diversified to decrease selection pressure and avoid the spread of new mutations. Knowledge of TSR background is essential, so the monitoring and testing of resistance *L. rigidum* in Maghreb countries (Morocco, Tunisia, including Algeria) is crucial. The use of next-generation sequencing will help to identify and quantify TSR status in the region with subsequent mapping. Integrated weed management, including the appropriate use of herbicides, should be adopted, especially in regions where cereal monocropping is highly abundant, in order to minimize the selection pressure and promote enhanced metabolism. Studying NTSR mechanisms evolved *L. rigidum* in cereal field populations, and management of this type of resistance in Maghreb is essential for the performance of chemical control in the future.

## Data availability statement

The original contributions presented in the study are publicly available. This data can be found here: https://www.ncbi.nlm.nih.gov/bioproject/PRJNA1045233/.

## Author contributions

ZE-M: Methodology, Data curation, Formal analysis, Investigation, Supervision, Writing – original draft. PK: Methodology, Supervision, Writing – review & editing. KH: Conceptualization, Methodology, Supervision, Writing – review & editing. AE: Methodology, Writing – review & editing. JS: Conceptualization, Supervision, Writing – review & editing.
